# Medical, Surgical, and Hygienic Exhibition

**Published:** 1898-06-11

**Authors:** 


### MEDICAL, SURGICAL, AND HYGIENIC EXHIBITION.

The second annual exhibition of the Medical, Surgical, and Hygienic Exhibitors' Association was successfully opened on Tuesday, May 31st, at the Queen's Hall, Langham Place, W., and was so great a success that it will probably become an annual institution.

One of the first stalls to attract attention on entering the vestibule was Camwal's exhibit of aërated waters which was well worth a visit.

Vimbos (Limited) had an attractive stall, at which the various "Vimbos" preparations were shown, the dainty samples of lozenges for cyclists being much appreciated.

A. Boake, Roberts, and Co. (Limited) were exhibiting a new disinfectant, called "Lozar," which is claimed to be more powerful than carbolic acid. ½ per cent. solution being regarded as a thoroughly efficient antiseptic and disinfectant.

Anderson, Anderson, and Anderson (Limited).—At this firm's exhibit was to be seen a fine display of water beds, cushions, and pillows, not only for sale but for hire. These great comforts can be obtained from Messrs. Anderson by poor patients for the sum of 2s. per week. Special notice was drawn to the improvements in their waterproof operation aprons and germ-proof clothing for use in infectious hospitals. This firm supplied most of the water-beds used in the recent Maidstone epidemic.

Domen Belts Company.—The main features at this stall were corsets with belt attachment, and belts for various women's ailments, support without pressure being the end aimed at.

Mayer and Meltzer showed instruments for intestinal surgery, among them being Mr. Collier's tubes for draining the intestines, Mr. Jackson Clarke's rubber anastomosis bobbins, and Haltead's supports for bowel and bile duct, as well as many new patterns of instruments for general surgery.

John Weiss and Son.—As one of the leading firms of surgical instrument makers, their stand naturally attracted considerable attention. Amongst other things was a model of an improved operating table; an automatic thermo-cautery, which does away with all india-rubber parts, was shown; as well as Dr. Fisher's patent knee-cap, and cases of sterilizable instruments made according to the most recent methods.

The Rebman Publishing Company (Limited).—This firm's medical publications were displayed to advantage. Their medical hand atlases attracted considerable attention.

The Maltine Manufacturing Company (Limited) and Carnrick and Co. (Limited).—These united firms displayed to advantage their coca wine and Carnrick's liquid food or peptonoids amongst other preparations, the stall being quite a centre of attraction.

Evans and Wormull.—The stall of this old-established firm of instrument makers was fully equipped with all the latest and most approved patterns of instruments, hospital furniture, and general appliances for the operating theatres.

W. K. Stacy generally displayed wallets, instruments, &c. Stacy's registered stand for fitting in nurses' bags was noticed as a great improvement. The regulation bottles and jars are securely held in their places, whilst everything in the bag is easy of access. The stoppers of the bottles are kept secure by a simple frame to prevent leakage.

Welford and Sons (Limited).—Theirs was a very prettily got-up stand, representing a model dairy, showing their well-known bottled milks.

Newton, Chambers, and Co. (Limited) were to the fore with their "Izal" disinfectants and preparations. The new "Izal" vaporiser, used in the treatment of phthisis, croup, whooping cough, &c., attracted a good deal of interest. The investigations made into this method give every hope of achieving satisfactory results in the treatment of the diseases mentioned. We observe that the Government has extensively adopted "Izal" for use in the military hospitals and in the navy.

Scott and Browne (Limited).—This firm's stall presented a creditable appearance, medical men and nurses manifesting keen interest in the Scott's Emulsion of Cod Liver Oil with Glycerine and Hypophosphites. Upon the stand was a pyramid surmounted by a life-sized figure of a Norwegian fisherman taken from life carrying a cod fish weighing 156 lbs.

Ronuk (Limited).—This company exhibited a specimen oak and teak floor, polished entirely with "Ronuk," and was much admired. This preparation seems to be admirably adapted for hospital ward floors.

Southall Bros. and Barclay.—Great interest was taken in this firm's surgical pads, as well as their splendid sanitary towels, and the new special absorbent material used in the manufacture of these articles.

The Sanitas Company (Limited).—In addition to the various "Sanitas" preparations, prominence was given to the "Sanitas" antiseptic surgical dressings and a new antiseptic called "Okol," which has been recently introduced.

The Milk Preparations Company, of Brentwood, Essex, displayed a representative collection of their high-class specialities in the "Sap" brand milk diets.

Ingram and Royle.—This firm exhibited the frequently prescribed "Hunyadi János," "Carlsbad," and "Vichy" waters, pastiles, salts, &c.

Callard and Co.'s exhibit consisted of diabetic breads, biscuits, and cakes, jellies, chocolates, &c., which they claim to be as palatable as the ordinary confectioners' article, but free from carbohydrates, thus pleasing the patient and satisfying the physician.

Cozenza and Co.'s stand, exhibiting "Maggi's

Consommé," drew the attention of numbers of those present. This successful preparation is now supplied to members of the Royal Family.

The Berkefeld Filter Company showed their germ proof rapid filters as fitted to the ordinary service pipe in households, as well as different forms of travelling filters, and the Berkefeld Aseptic Irrigator for supplying sterilised water of regulated temperature for use in surgical operations.

Friern Manor Dairy Farm (Limited) exhibited Dr. Gaertner's process of humanising milk. As showing the success of this company, the sales alone, according to reports from Continental centres, totalled over a million bottles in 1897.

Stower's Lime Juice Cordial stand was well ladened with this wholesome beverage, and presented quite a hearty and refreshing appearance.

J. Defries and Sons.—Amongst other various types of their well-known filters, this firm exhibited a new exhaust filter which takes the place of a pressure filter. A special feature of the stall was Dr. Scurfield's patent ventilation indicator, to register the amount of carbonic acid gas in a sick-room. This indicator is in use in several hospitals.

Idris and Co. (Limited) exhibited general aërated beverages, making a specialty of medical salts in carbonated distilled water, put up in syphons graduated in wineglasses.

Cadbury Bros.—This old-established firm's stand was most inviting, as a cup of their freshly-made cocoa was always obtainable. The main feature of their cocoa is absolute purity.

*(To be continued.)*

